# Detection of Newly Described Astrovirus MLB1 in Stool Samples from Children

**DOI:** 10.3201/eid1601.091120

**Published:** 2010-01

**Authors:** Krisztián Bányai, Edina Meleg, Paschalina Moschidou, Vito Martella

**Affiliations:** Veterinary Medical Research Institute, Budapest, Hungary (K. Bányai); University of Pécs, Pécs, Hungary (K. Bányai E. Meleg); University of Bari, Valenzano, Italy (P. Moschidou, V. Martella)

**Keywords:** Astrovirus MLB1, Mexico, enteric virus, children, stool samples, viruses, letter

**To the Editor:** We read with interest the article by Finkbeiner et al. describing an epidemiologic survey of newly described astrovirus MLB1 (AstV-MLB1) conducted in the United States in 2008 (*1*). This study was an extension of recently published reports of characterization of AstV-MLB1 from a fecal sample obtained in Australia in 1999 (*2*,*3*). These studies provide evidence of a divergent group of astroviruses and their etiologic association with human disease.

However, the occurrence of a MLB1-like AstV in humans has already been documented. Walter identified a novel AstV in an 8-month-old child with diarrhea in Mexico in 1991 (*4*). In that study, Walter screened fecal samples for AstVs by using a variety of techniques. Sequencing of selected PCR products identified a unique virus that had typical AstV morphologic appearance but was nonreactive with human AstV-specific monoclonal or polyclonal antibodies. Phylogenetic analysis of fragments of open reading frame 1a (ORF1a) and ORF2 genome regions of this virus strain (M3363) showed that it was only distantly related to other mammalian AstVs, including human AstVs (*4*). This sequence divergence from canonical human AstVs suggested that M3363 might have been transmitted from an animal reservoir (*4*,*5*).

When we reanalyzed ORF1a of M3363, we found that this strain was actually an MLB1-like AstV with >98% amino acid similarity to prototype and strains from the United States (Figure) that dated back to 1991. The fact that such closely related viruses were found in a scattered temporal and spatial pattern in children may indicate that MLB1-like AstVs represent a true human enteric virus, which was probably overlooked in the absence of adequate diagnostic reagents and protocols.

**Figure:**
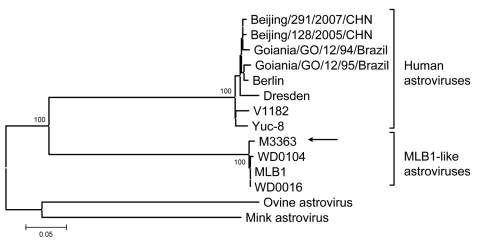
Neighbor-joining tree based on partial sequences of open reading frame 1a protein of human and animal astroviruses. Amino acid sequences of the Mexican M3363 strain (arrow) were obtained from Walter (*4*); other sequences were obtained from GenBank. Bootstrap values >90 are indicated. Scale bar is proportional to genetic distance and indicates nucleotide substitutions per site.
