# Supplementary material for: Detection of Newly Described Astrovirus MLB1 in Stool Samples from Children
Source: Emerg Infect Dis. 2010 Jan;16(1):169–70. doi: 10.3201/eid1601.091120 (PMC2874377; doi:10.3201/eid1601.091120)
Supplement: Related texts

# Letter

**DOI:** 10.3201/eid1601.091563

**Published:** 2010-01

**Authors:** Stacy R. Finkbeiner, Binh-Minh Le, Lori R. Holtz, Gregory A. Storch, David Wang

**Affiliations:** Washington University School of Medicine, St. Louis, Missouri, USA

## In Response

We thank Bányai et al. (*1*) for drawing attention to the unpublished data of Walter (*2*), which was not part of the peer-reviewed literature at the time we described the complete genome of astrovirus MLB1 (*3*) or when we described our epidemiologic survey of stools collected in St. Louis, Missouri, USA (*4*). The results of Walter extend the known geographic range of astrovirus MLB1 to include Mexico, thus supporting our recent proposal that astrovirus MLB1 is likely to be globally widespread (*4*). We look forward to including the partial sequence generated by Walter in future analyses of astrovirus MLB1 genetic diversity. We strongly encourage Bányai et al. to submit their sequence data to any of the publicly accessible and searchable international sequence databases such as GenBank, the European Molecular Biology Nucleotide Sequence Database, or the DNA Database of Japan so that it can be readily accessed by the scientific community.

## References

[1] Bányai K, Meleg E, Moschidou P, Martella V. Detection of newly described astrovirus MLB1 in stool samples from children [letter]. Emerg Infect Dis. 2010;16:169. 10.3201/eid1601.09112020031079PMC2874377

[2] Walter JE. Genetic characterization of astroviruses associated with diarrhea among children in a periurban community of Mexico City, 2002 [doctoral dissertation]. Pecs (Hungary): Medical School, University of Pecs.

[3] Finkbeiner SR, Kirkwood CD, Wang D. Complete genome sequence of a highly divergent astrovirus isolated from a child with acute diarrhea. Virol J. 2008;5:117.1885403510.1186/1743-422X-5-117PMC2576171

[4] Finkbeiner SR, Le BM, Holtz LR, Storch GA, Wang D. Detection of newly described astrovirus MLB1 in stool samples from children. Emerg Infect Dis. 2009;15:441–4 . 10.3201/eid1503.08121319239759PMC2666294
